# The role of the C-terminal helix of U1A protein in the interaction with U1hpII RNA

**DOI:** 10.1093/nar/gkt326

**Published:** 2013-05-22

**Authors:** Michael J. Law, Diane S. Lee, Charlene S. Lee, Paul P. Anglim, Ian S. Haworth, Ite A. Laird-Offringa

**Affiliations:** ^1^Department of Biochemistry and Molecular Biology, Keck School of Medicine, University of Southern California, Los Angeles, CA 90089, USA, ^2^Department of Surgery, Keck School of Medicine, University of Southern California, Los Angeles, CA 90089, USA and ^3^Department of Pharmacology and Pharmaceutical Sciences, School of Pharmacy, University of Southern California, Los Angeles, CA 90089, USA

## Abstract

Previous kinetic investigations of the N-terminal RNA Recognition Motif (RRM) domain of spliceosomal A protein of the U1 small nuclear ribonucleoprotein particle (U1A) interacting with its RNA target U1 hairpin II (U1hpII) provided experimental evidence for a ‘lure and lock’ model of binding. The final step of locking has been proposed to involve conformational changes in an α-helix immediately C-terminal to the RRM domain (helix C), which occludes the RNA binding surface in the unbound protein. Helix C must shift its position to accommodate RNA binding in the RNA–protein complex. This results in a new hydrophobic core, an intraprotein hydrogen bond and a quadruple stacking interaction between U1A and U1hpII. Here, we used a surface plasmon resonance-based biosensor to gain mechanistic insight into the role of helix C in mediating the interaction with U1hpII. Truncation, removal or disruption of the helix exposes the RNA-binding surface, resulting in an increase in the association rate, while simultaneously reducing the ability of the complex to lock, reflected in a loss of complex stability. Disruption of the quadruple stacking interaction has minor kinetic effects when compared with removal of the intraprotein hydrogen bonds. These data provide new insights into the mechanism whereby sequences C-terminal to an RRM can influence RNA binding.

## INTRODUCTION

RNA recognition motifs (RRMs) are the most commonly found RNA-binding domain in eukaryotes and are implicated in many critical RNA–protein interactions in the cell (1–4). RRM-containing proteins interact with their RNA targets with dynamics that reflect their biological function. For example, RNA–RRM interactions that play a structural role in the ribosome must form more stable complexes than RNA–RRM interactions required for pre-mRNA processing (1–4). Studies focused on characterizing the dynamics of RNA–RRM interactions can provide insight into the mechanistic basis underlying these interactions.

**Figure 1.**
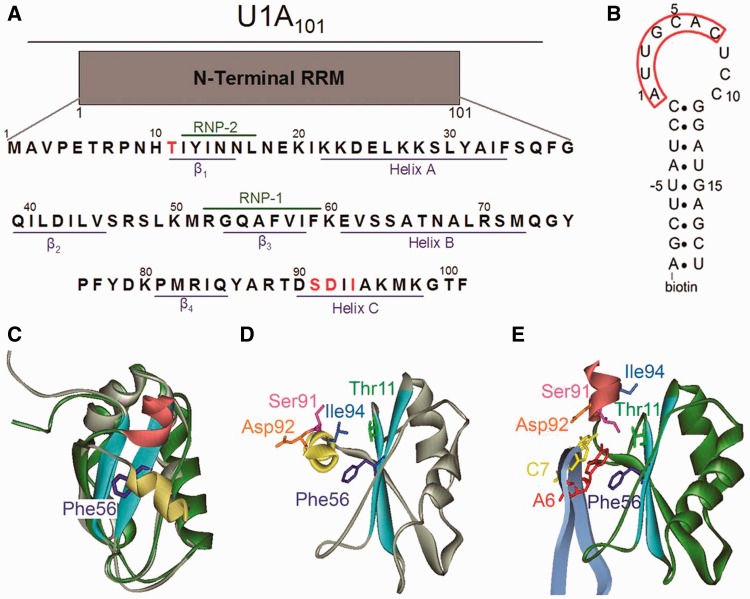
Representation of free U1A and U1A–U1hpII complex. (**A**) Primary amino acid sequence of the U1A construct used in this study. Structural features of the RRM domain are shown in blue, conserved RNP regions are marked in green and amino acids mutated in this study are shown in red. (**B**) Sequence of the U1hpII RNA used for the biosensor analyses. Nucleotides U-5 to G15 are identical to the wild-type sequence. The seven loop nucleotides key for binding U1A are boxed in red. The molecule is biotinylated at its 5′ end for attachment to the Biacore surface. (**C**) Overlay of NMR structure of unbound U1A (gray, pdb ID 1FHT, model 1 (20)) and U1A in its co-crystal with U1hpII (green, pdb ID 1URN (20,21)). The gray and green colors are chosen to show the similarity of the overall fold of the RRM domains in the two structures, which show strong overlap. Conserved RNP regions are shown in light blue, the position of helix C in the free protein is shown in yellow, and the position of helix C in the co-crystal is shown in red. (**D**) Position of the mutated amino acids in the free U1A NMR structure (1FHT; 20). Conserved RNP regions are shown in light blue, helix C is shown in yellow and amino acids mutated are shown in various colors. (**E**) Position of amino acids of interest in U1A–U1hpII co-crystal structure (1URN; 21). Conserved RNP regions are shown in light blue, helix C is shown in red and amino acids mutated are shown in various colors.

RRMs are characterized by a β-α-β-β-α-β secondary structure that results in the formation of an antiparallel β-sheet comprising the RNA-binding platform (5). The most conserved regions of the RRM are centrally located in the β-sheet, containing stretches of 8 and 6 amino acids that are referred to as the ribonucleoprotein consensus sequences 1 and 2 (RNP-1 and -2), respectively (Figure 1A) (2–4,6,7). RNP-1 and -2 are critical for high-affinity RNA binding by RRM-containing proteins, as evidenced by the dramatic loss in affinity on their mutation (8–14). In contrast, the distinct binding ‘specificity’ of different RRM-containing proteins is strongly influenced by the more variable regions of the domain.

Structural analyses of RRM-containing proteins have indicated that regions C-terminal to the RRM domain can be important for RNA-binding affinity and specificity by increasing the number of contacts between the protein and its RNA target (15). The RRM-containing protein CstF-64 contains a C-terminal α-helix that unfolds on RNA binding, while nucleolin, HuD, poly(A) nuclear cap binding protein and sex lethal all contain α-helices that become ordered on RNA binding (16–18). U1A, the A protein of the U1 small nuclear ribonucleoprotein particle (snRNP), which binds to hairpin II in the U1 small nuclear RNA (U1hpII) through its N-terminal RRM domain, also contains a C-terminal α-helix (helix C). NMR and radiographic crystallography studies have shown that helix C consists of residues Asp90 to Lys98 (Figure 1) (19,20). In the absence of RNA, the helix lies in a ‘closed’ conformation, occluding the RNA binding surface. In this conformation, helix C residues Ile93, Ile94 and Met97 interact with RNP-1 residues Phe56 and Ile58, as well as β-strand 2 residue Leu44, forming a hydrophobic core (Figure 1A, C and D) (20). The side chains of Ala95, Lys96 and Lys98 are solvent exposed in the free (19,20) and bound (21) protein.

To interact with U1hpII, RNP-1 must be exposed. This occurs through a shift in the position of helix C relative to the RNA-binding region by >90° to an ‘open’ protein conformation (Figure 1C) (20,21). To accommodate U1hpII, the main-chain torsion angles of Thr89, Asp90 and Ser91 must change, which results in a repositioning of the hydrophobic core (20,21). In the U1A–U1hpII complex, the new hydrophobic core is defined by Ile93 and Ile94 contacting His10, Leu41, Ile58 and Val62 (21). The repositioned helix C also makes a new intraprotein contact through a hydrogen bond between Ser91 and Thr11. In addition, Ser91 forms a hydrogen bond with N1 of RNA nucleotide A6 (21). Helix C is also involved in a quadruple stacking interaction in which RNA bases A6 and C7 are sandwiched between Asp92 and Phe56 (Figure 1B and E) (21,22). This stack involves hydrophobic contacts and pi electron overlap between the aromatic ring, RNA bases and the carboxylate group of Asp92. Lastly, the main chain amino group of Asp92 makes a hydrogen bond to N3 of RNA base C7 (21). The relative contributions of the interactions of the repositioned helix to complex formation and stability remain to be fully investigated.

Biochemical, structural and computational analyses of the N-terminal region of U1A containing RRM1 (amino acids 1–101, herein referred to as U1A) suggest that helix C is dynamic and may be stable in both the ‘closed’ and ‘open’ conformations independent of interactions with RNA targets (23–26). However, recent time-resolved fluorescence anisotropy experiments and molecular dynamics simulations of the free protein detected only an ∼20° angular motion of helix C (25,27,28). It is unclear whether these observations were limited by the experimental conditions. Combined with equilibrium binding analyses, these prior studies suggest a model in which U1A helix C stabilizes the unbound protein but also plays an important role in locking onto the RNA target in the complex (5,12,21,29–31). However, kinetic data testing this dynamic function of helix C has not been available to date.

Here, we use a surface plasmon resonance-based biosensor (Biacore) to analyze the role of helix C in the interaction with U1hpII. Biacore allows the collection of high-quality kinetic measurements that facilitate the dissection of features important for complex formation (through measurements of association rates) versus complex stability (through measurements of dissociation rates). Previous kinetic analyses of the U1A–U1hpII interaction have led to the development of a ‘lure’ and ‘lock’ model in which well-placed positively charged amino acids bring U1A into proximity with U1hpII, and then close-range interactions lock the complex into place (14,32,33). In our stepwise model describing locking, the positioning of helix C was suggested as the final step of the interaction, likely contributing to the unusual stability of the complex (14). Here, we probe different aspects of helix C to clarify its role in controlling access to the RNA-binding surface and locking the RNA in place.

## MATERIALS AND METHODS

### Construction of U1A mutants, protein purification and circular dichroism analysis

U1A mutagenesis, production and quantification were as previously described with the following modifications (32,33). To focus on helix C function, we moved the hexahistidine tag from the C- to N-terminal end of the U1A fragment, and detected no statistically significant difference in U1hpII binding kinetics between the two proteins. All mutations were confirmed by sequencing. Circular dichroism (CD) spectra were collected from U1A and U1A90. The proteins were exchanged into a 10 mM sodium phosphate buffer pH 7.0, were diluted to 12 μM in the same buffer and placed in a Jasco J-810 CD Spectropolarimeter. Spectra were read from 195 nm to 260 nm using 1 mm pathlength quartz cuvettes. The baseline (buffer only) measurement was subtracted from each of the protein spectra before they were overlaid.

### Biosensor analysis

**Figure 2.**
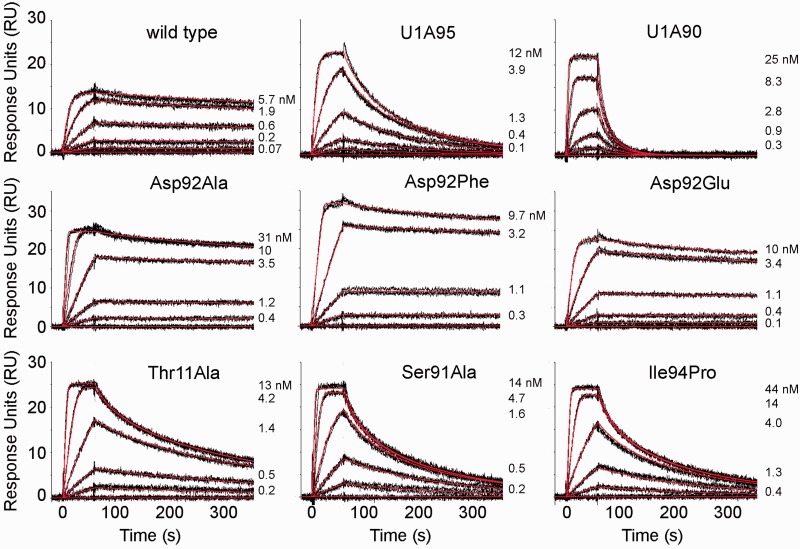
Sensorgrams showing kinetic analyses of wild-type U1A and protein mutants interacting with U1hpII. Top row: Wild-type U1A and helix C truncation mutants. Middle row: Asp92 mutants. Bottom row: Thr 11, Ser 91 and Ile94 mutants. Protein concentrations injected are as indicated. Black lines represent triplicate injections, which were performed in random order over a U1hpII surface. Association was monitored for 1 min followed by a 5 min dissociation phase. Red lines represent the global fit of data sets using CLAMP (35). Kinetic parameters for the experiments are shown in Table 1.

Binding experiments were performed on a Biacore 2000 instrument as previously described (32,33). To inhibit non-specific protein–RNA interactions, running buffer included 125 µg ml^−1^ yeast tRNA (Roche, IN) and 62.5 µg ml^−1^ acetylated bovine serum albumin (New England Biolabs, MA). To provide an optimal comparison of the results obtained from all different U1A mutants, we prepared two surface densities on the sensor chip, an intermediate density RNA surface (100–125 resonance units) that would yield sufficient signal, even when proteins with lower affinities were used, and a low-density RNA surface (35–50 resonance units). Proteins were serially diluted in running buffer to the concentrations indicated in Figure 2. Samples with different concentrations of protein were injected in random order, and every injection was performed in triplicate within each experiment. All experiments were done four to six times. Double referencing was performed in which all samples were run over an unmodified sensor chip surface, allowing background noise subtraction (34). Data was processed using Scrubber and analyzed using CLAMP XP (35) and a simple 1:1 Langmuir interaction model with a correction for mass transport (36). Statistical significance was tested using the Student’s *t*-test; equal and unequal variance for the samples was determined using the *F*-test.

### Molecular dynamics and solvation calculations

Molecular dynamics simulations were performed in AMBER10 with solvation of the protein–RNA complex in a periodic box of TIP3P water molecules at 298 K with a time step of 1 fs and a vdW cutoff of 10 Å. Chains B and Q of the U1A-U1hpII radiographic structure (PDB ID 1URN; (21)) were used as the starting point for the calculations. Solvation of the ‘interface' between helix C and the RRM was calculated for the wild type and two mutated U1A proteins using WATGEN (37,38). Calculations were performed on all 43 complexes of the ensemble of NMR structures in PDB ID 1FHT (20).

## RESULTS

### Truncation or disruption of helix C increases the rate of complex formation while destabilizing the U1A–U1hpII complex

The role of helix C in the interaction of U1A (Figure 1A) with U1hpII (Figure 1B) was first investigated by performing kinetic analyses using U1A proteins in which the helix was truncated halfway at amino acid 95 (U1A95) or completely removed by terminating the protein after amino acid 90 (U1A90). Previous equilibrium-based analyses have indicated that the affinity of U1A95 for U1hpII is decreased by ∼20- to 45-fold (29,31,39), while truncation to U1A90 decreased the affinity for U1hpII by up to three orders of magnitude (29). These observations demonstrate the importance of the helix in the interaction with U1hpII, but do not define the underlying cause for the losses in affinity. CD spectra (Supplementary Figure S1) indicate that the fold of RRM1 is maintained in U1A90 compared with U1A and that the reduced affinity of U1A90 for U1hpII is not caused by perturbation of the RRM structure owing to complete removal of helix C.

**Figure 3.**
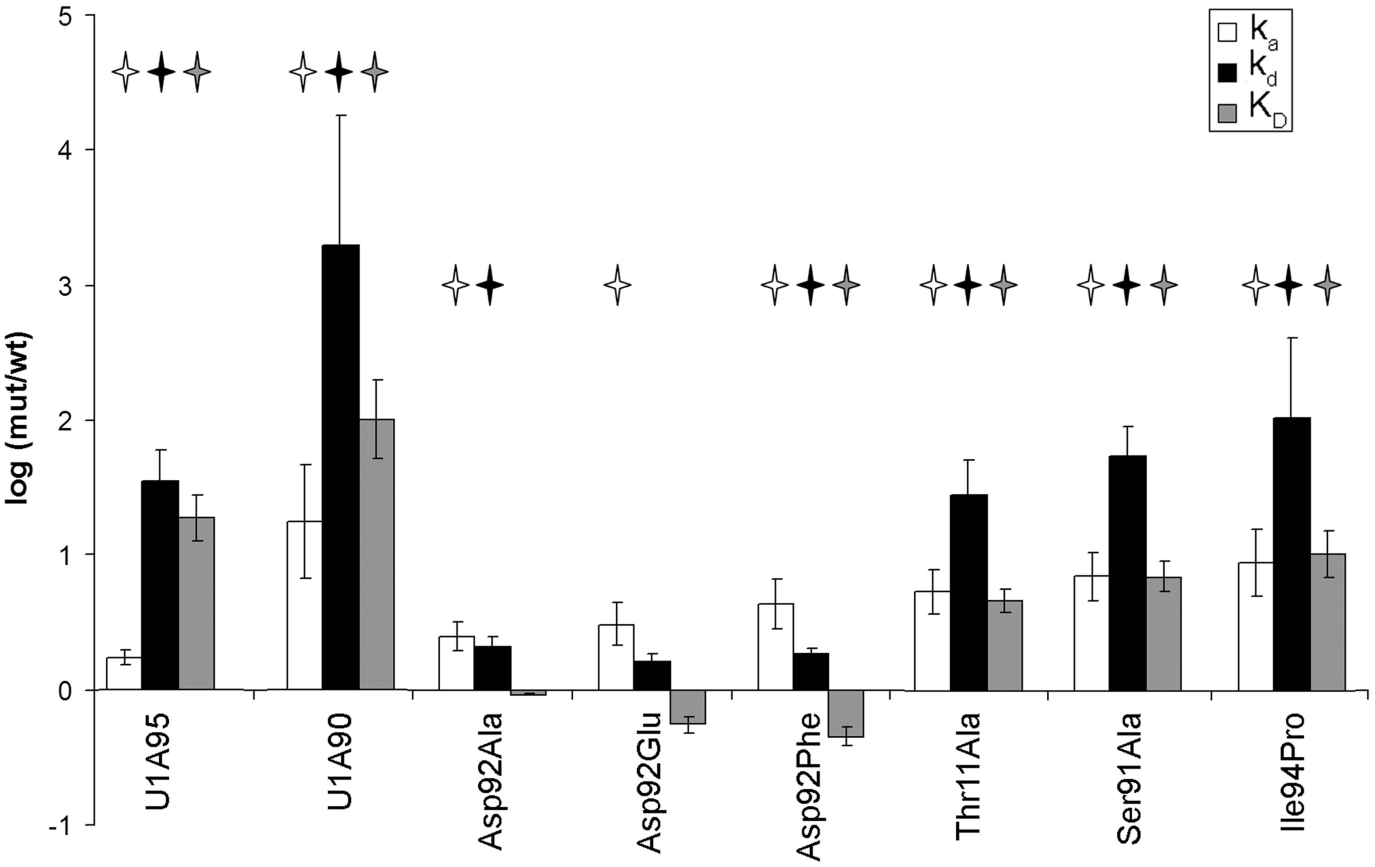
Effects of C-terminal helix mutations on *k*_a_, *k*_d_ and *K*_D_. To visualize the differences between mutants and wild-type protein, we plotted the logarithm of wild-type/mutant values for *k*_a_ and mutant/wild-type values for *k*_d_, and *K*_D_. Error bars indicate the standard error of the mean, while stars represent values that are significantly different from wild type.

Kinetic analysis of U1A95 showed a modest but statistically significant increase in the association rate (1.7-fold increase), which was offset by a ∼35-fold loss in complex stability (Table 1 and Figures 2 and 3). This resulted in a net loss of ∼20-fold in affinity, in agreement with published studies (29). Further deletion of amino acids 91–95 caused more dramatic kinetic effects; U1A90 showed a statistically significant 18-fold increase in association rate, with a ∼2000-fold loss in complex stability (Table 1 and Figures 2 and 3), resulting in a net affinity loss of two orders of magnitude, also consistent with previous reports (29,31,39). In both truncation mutants, the association rate increased, consistent with occlusion of the RNA-binding surface by helix C in the unbound protein. Importantly, the association rate increase of U1A90 is significantly larger than that of U1A95, indicating that the remaining fragment of helix C in U1A95 still covers part of the RNA binding surface, slowing complex formation (Figure 1C).

**Table 1.** Kinetic parameters of interactions between U1A mutants and U1hpII

Protein	*k*_a_ (M^−1^s^−1^)	Fold increase	*k*_d_ (s^−1^)	Fold increase	*K*_D_ (M)	Fold decrease
Wild type	1.3 ± 0.3 × 10^7^		5.2 ± 0.6 × 10^−4^		4.4 ± 0.6 × 10^−11^	
U1A95	2.3 ± 0.3 × 10^7^	1.7	1.8 ± 0.2 × 10^−2^	35	8.2 ± 0.2 × 10^−10^	19
U1A90	2.3 ± 0.6 × 10^8^	18	1.0 ± 0.3 × 10^0^	2000	4.5 ± 0.3 × 10^−9^	100
Ile94Pro	1.2 ± 0.2 × 10^8^	9.2	6 ± 1 × 10^−2^	100	5 ± 0.5 × 10^−10^	10
Asp92Glu	4 ± 1 × 10^7^	3.1	**9 ± 2 × 10^−^^4^**	1.7	**2 ± 0.4 × 10^−11^**	0.6
Asp92Ala	3.3 ± 0.6 × 10^7^	2.5	1.1 ± 0.2 × 10^−3^	2.1	**4.1 ± 0.9 × 10^−^^11^**	0.9
Asp92Phe	6 ± 1 × 10^7^	4	1.0 ± 0.1 × 10^−3^	1.9	2.0 ± 0.3 × 10^−11^	0.5
Thr11Ala	7.1 ± 0.7 × 10^7^	5.5	1.5 ± 0.2 × 10^−2^	28	2.1 ± 0.1 × 10^−10^	4.8
Ser91Ala	9.3 ± 0.6 × 10^7^	7.2	2.9 ± 0.2 × 10^−2^	55	3.1 ± 0.04 × 10^−10^	7.0

Average and standard error of the mean are given. All values are statistically significantly different from wild-type U1A except those in bold. The *K*_D_ was calculated for each experiment from the globally fitted *k*_a_ and *k*_d_ (*K*_D_ = *k*_d_/*k*_a_) and was averaged based on four or more experiments. Ratios for the fold change are approximated and are generally shown as a number >1 (mut/wt).

To examine the effect of disrupting helix C, rather than removing it, we introduced a proline at position 94 (Ile94Pro). Ile94 makes a backbone hydrogen bond to Ser91, forming the first turn of the helix, and its mutation to proline should disrupt the short helix C, as predicted by a helix propensity scale for globular proteins (40). The mutant exhibited an ∼9-fold increase in the association rate, paired with a 100-fold loss in complex stability, resulting in a 10-fold net loss in affinity (Table 1 and Figures 2 and 3). Molecular dynamics simulations of Ile94Pro in complex with U1hpII indicate that interactions between Ser91 and Ala95 and between Asp92 and Lys96 are weaker in the mutant relative to wild type (Supplementary Figure S2). Therefore, the large loss in complex stability we observe in the Ile94Pro mutant is most likely due to disruption of the conformation of helix C.

The much greater loss in stability of the U1A90–U1hpII complex relative to that of U1A95 or the Ile94Pro mutant points to a major role for amino acids 91–94 in stabilizing the protein–RNA complex. Amino acids in this region form multiple types of intra- and intermolecular interactions, including the quadruple stack contributed by Asp92, the hydrogen bond from Asp92 to C7 and the hydrogen bond between Ser91 and Thr11. We undertook site-directed mutagenesis to examine the contributions of these amino acid residues in more detail.

### Removal of the quadruple stack with Asp92 has minor kinetic effects

We investigated the kinetic contribution of the quadruple stacking interaction in which Asp92 is connected to RNP-1 residue Phe56 via bases A6 and C7 of the U1hpII RNA loop (Figure 1E) (21). Molecular modeling has suggested that the major loss of affinity of helix C truncation mutants for U1hpII is due to the loss of the quadruple stack (22). In free U1A, Asp92 is solvent exposed and does not interact with other parts of the RRM domain (Figure 1D), suggesting that its mutation should not influence U1A–U1hpII complex formation (19,20,26). While Asp92 contributes both a stacking interaction and a hydrogen bond to the interaction with U1hpII, the hydrogen bond is mediated through the main chain amino group. Therefore, Asp92 mutations would not be predicted to prevent formation of this hydrogen bond, allowing direct assessment of the stacking interaction. To determine if the reduced binding in the U1A90 mutant is due to loss of the quadruple stack, we mutated Asp92 to Glu, Ala or Phe (Figure 2). Mutation to Glu is a conservative replacement serving as a control, mutation to Ala should prevent the formation of the quadruple stack and mutation to Phe should restore the stacking interaction, possibly even strengthening it.

The Asp92Glu, Ala and Phe mutants all showed statistically significant increases in *k*_a_ of 2.5- to 4-fold. (Table 1 and Figures 2 and 3), suggesting that even a conservative mutation to Glu can destabilize the closed position of helix C. Complex stability was not significantly affected in the Asp92Glu mutant, and was modestly but significantly decreased (∼2-fold) for both Asp92Ala and Asp92Phe (*P* < 0.05; Table 1 and Figures 2 and 3). This suggests that the contributions of the Asp92 sidechain to complex stability are modest, and that loss of the ‘lid’ of the quadruple stack does not explain the dramatic destabilization of the complex by truncation of the C-terminal helix. These data suggest that the role of Asp92 in stabilizing the complex requires its main chain H-bond, which would be lost in the U1A90 truncation. We further addressed the hydrogen bonding network in U1A by examining intraprotein and intermolecular hydrogen bonds.

### Disrupting the U1A–U1hpII hydrogen bonding network results in helix unpacking and complex instability

We investigated the contribution of the intraprotein hydrogen bond between Thr11 and Ser91 that is formed in the U1A–U1hpII complex (Figure 1E). Thr11 is located in the β-1 strand adjacent to RNP-2 amino acid Tyr13, a residue that provides stacking interactions with RNA bases and is important for both complex formation and stability (14). In the U1A–U1hpII complex, Ser91 forms two hydrogen bonds, one to N1 of A6 in the loop of U1hpII and the other to Thr11 (20,21). Previous equilibrium-based experiments indicated that mutation of either Thr11 or Ser91 is deleterious to RNA binding (29). To kinetically assess the importance of these hydrogen bonds, we individually mutated Thr11 and Ser91 to Ala (Thr11Ala and Ser91Ala) and examined binding to U1hpII.

Complex stability was considerably reduced for the Thr11Ala and Ser91Ala mutants, with 28- and 55-fold increases in *k*_d_, respectively (*P* < 0.05, Table 1 and Figures 2 and 3). This indicates that the hydrogen bond between the two residues is important for locking the C-terminal helix onto the RNA. In addition to defects in complex stability, both Thr11Ala and Ser91Ala also showed a statistically significant increase in the association rate of 5.5-fold and ∼7-fold, respectively (Table 1 and Figures 2 and 3). This suggests that mutations of these residues affect access of the RNA to the binding surface, and confirms suggestions from prior studies that these residues could play a role positioning helix C in free U1A (20,29).

To test whether these mutations affect helix C positioning before interacting with U1hpII, we undertook computational analyses to examine solvation of the mutants. We used an algorithm (WATGEN) for interface solvation that was developed in our laboratory (37,38). The solvation shell filling the ‘interface' between helix C and the RRM was calculated for the wild-type and mutated proteins for each of the 43 NMR-derived models (PDB ID: 1FHT) of unbound U1A (20). Water molecules bridging between Thr11 and Ser91 were found in 36 of the 43 models for the wild-type protein, with most bridges occurring between the Thr11 side chain OH and the Ser91 side chain OH or backbone NH (Supplementary Table S1). The loss of Thr11 OH in the Thr11Ala mutant eliminated the water bridge in all except five models (Supplementary Table S2). Water bridges were retained in 24 of the 43 models for the Ser91Ala mutant, mainly due to bridging between Thr11 OH and Ala91 NH (Supplementary Table S3). The decrease in water bridges between residues 11 and 91 was significant in the two mutants (*P* < 0.0001 and *P* < 0.005, respectively, by Student’s *t*-test). An illustration of the solvent calculation is shown in Supplementary Figure S3. The bridging water molecule in the wild-type protein (indicated by the arrow in Supplementary Figure S3B) is lost in the mutated proteins (Supplementary Figure S3C and D). Loss of this water bridge and further disruption of the solvation shell between helix C and the RRM may result in easier movement of helix C, as suggested by the increased *k*_a_ values for interaction of U1hpII with the mutated proteins.

## DISCUSSION

The N-terminal region of U1A, containing RRM1, and its interaction with its RNA target in the U1snRNA, U1hpII, has served as a powerful model for defining the structure and mechanism of RRM-containing proteins interacting with their RNA targets. The U1A–U1hpII interaction is among the strongest non-covalent interactions described, and determining its molecular basis is of interest with respect to both understanding the U1A–U1snRNP complex as well as understanding how tight biological interactions can be formed and maintained.

Similar to many other RRM proteins, in U1A an alpha helix lies C-terminal to the RNA binding platform (helix C). This helix is thought to participate both in complex formation and stability. Here, we dissected its two roles. We find that helix C is of key importance in stabilizing the RNA–protein complex; the dissociation rate increases by three orders of magnitude with helix C removal. The biggest contribution of helix C to complex stability was not, as expected, the quadruple stack terminated by Asp92. We found that the role of Asp92 in this interaction is largely dispensable for a stable U1A–U1hpII complex. Mutations of Asp92 to Glu, Ala or Phe exhibited minor defects in complex stability (∼2-fold), but showed more substantial increases in the rate of complex formation (2.5- to 4-fold), so that net affinity was unaffected or even slightly improved. Our previous kinetic analysis of Phe56 mutants has indicated that loss of this aromatic residue results in a 6500-fold reduction in complex stability (14). Thus, it would appear that stacking of A6 on Phe56 is the major driver of complex formation and that this interaction forms even in the absence of Asp92, suggesting that the Asp92 sidechain plays a minor role in locking together the U1A–U1hpII complex. These results point to other amino acids within the helix as being the major contributors to the losses in complex stability seen in the U1A90 truncation. Both the Thr11Ala and Ser91Ala mutations showed pronounced increases in the *k*_d_. In the Thr11Ala mutant complexed with U1hpII, only the Thr11 to Ser91 hydrogen bond is lost, but in the Ser91Ala mutant, both the hydrogen bonds to Thr11 and to N1 of base A6 are lost. The merely 2-fold faster dissociation of Ser91Ala than Thr11Ala might suggest that the hydrogen bond to A6 plays a modest role in complex stability. However, experiments in which the N1 hydrogen donor of nucleotide A6 was mutated showed a 50-fold loss in affinity between U1A and the mutated RNA target (41), indicating that this interaction is highly relevant. This suggests that the Thr11 to Ser91 hydrogen bond plays a role in properly positioning Ser91 for its interaction with A6, and thus that disruption of the Thr11–Ser91 interaction also impedes the hydrogen bond to A6, so that the effects of both protein mutations are similar. Importantly, the loss in affinity with either of these mutations is substantially less than when RNA base A6 is mutated. This makes sense; when A6 is mutated, there is no compensatory increase in the association rate because accessibility of the protein to the RNA has not changed (see below).

In other RRM proteins, adjacent helices also assist in mediating stable protein–RNA complexes. Numerous other proteins that lack a C-terminal helix in the free protein increase the number of contacts between their RRM domain and their RNA target by forming a C-terminal helix on interaction with RNA (such as HuD, Pab, Sxl, CBP-20 and hnRNPA1) (16–18,42–46). These helices typically contribute to protein–RNA stability by making intra- and intermolecular H-bonds, similar to those in U1A–U1hpII complex.

Besides negatively affecting complex stability, helix C removal or disruption positively affects the association of U1A with U1hpII. The contradictory consequences of helix C perturbations mask its key role in stabilizing the RNA–protein complex when equilibrium measures are used to assess binding; they reduce the net effect of helix C perturbations on the affinity. This emphasizes the importance of kinetic studies when dissecting the role of structural elements in binding interactions. The largest increase in the rate of complex formation was observed when the helix was completely removed (U1A90), providing experimental confirmation that this helix occludes the RNA-binding surface. However, improved association was even observed in more subtle mutants. In both the Thr11Ala and Ser91Ala mutants, an association rate increase mitigated the effect of the loss of the intraprotein bond these residues provide in the complex, so that the net loss in affinity is <10-fold. Computational examination of the solvent network between helix C and the RRM indicated loss of bridging water molecules between Thr11 and Ser91 in both mutated proteins. This suggests that in these mutants, the helix is not packed as tightly against the RRM, which may allow easier association with U1hpII. These data indicate that solvation can influence the rate of association, while previous work has shown that ionic strength can affect the dissociation rate (47). These observations emphasize that factors other than direct protein–RNA interactions can play a role in determining both complex formation and stability.

C-terminal helices in other RRM-containing proteins appear to play diverse roles that contribute significantly to the function of these proteins. In U1A and proteins including hnRNP F, p14, U2AF, La protein and CstF-64, a preexisting C-terminal helix covers the RRM surface of the unbound protein, forming a hydrophobic core involving RNP amino acids. In hnRNP F, p14 and La protein, the helix remains in the ‘closed’ position because the RRM interacts with RNA via a non-canonical mechanism, without using the typical RNA-binding platform (48,49). Maintaining a ‘closed’ helix limits the types of RNA targets that these proteins can recognize, potentially playing a role in target discrimination (48–54). In an alternative mechanism, the occlusion of the RNA binding surface can be relieved by conformational change of the helix. This occurs in Cstf-64, when the helix unfolds and is lost in the protein–RNA complex (18). In the case of U1A, the C-terminal helix maintains its structure as it rearranges significantly to contribute to the protein–RNA complex. A possible function of the occlusion of the RNA binding surface by helix C might be to prevent association of non-target RNA, similar to the role of the helix in p14, La protein and CstF-64. In this model, only the correct target RNA could initiate the chain of events that displaces the helix from the RNA-binding surface.

In the above examples, a short alpha helix plays a role in inhibiting RNA contacts with the RNA-binding surface of the RRM. Accessory domains could lie at either side of RRMs and could come in many different forms. Examples of RRM autoinhibition exist in which a fully formed downstream RRM domain initially occludes the RNA binding surface, such as in HuD and U2AF65 (55,56). HuD contains three tandem RRM domains; removal of RRM3 increases the association rate of HuD with AU-rich RNA sequences, while resulting in an overall loss in RNA affinity (55), similar to the results observed on removal of helix C (U1A90). In U2AF65, two RRMs mediate interaction with RNA by forming two distinct conformations: an ‘open’ conformation in which the RNA binding surface is exposed to the RNA, allowing interactions with polypyrimidine tract RNAs, and a ‘closed’ conformation in which the RRMs interact with each other to inhibit long tracts of RNA from interacting with the RRM (56).

In addition to modulating access of U1hpII to the RNA-binding surface, the snug fit of U1A helix C onto the unbound RNA-binding platform might also improve the stability of the free protein. For example, in the analogous spliceosomal protein 65 K, a region N-terminal to the RNA-binding RRM forms two alpha helices in the unbound protein (57,58). Removal of these helices is deleterious to RNA binding, owing to a dramatic destabilization of the unbound protein (58). However, CD spectra of U1A and U1A90 indicated no effect of the absence of helix C on the RRM fold of recombinant U1A.

While the study of the U1A–U1hpII complex is of great interest owing to its remarkable stability, it should be noted that in nature, U1A does not bind to U1hpII in isolation; both are sections of larger molecules. The full-length U1A protein and the complete U1snRNA are components of the U1snRNP, which contains numerous other proteins (59). Thus, regions beyond helix C in U1A and interactions with other snRNP proteins may also influence binding to the RNA. Determining the kinetic sequence of events of particle assembly using the full proteins would be interesting and is an exciting challenge for the future.

## SUPPLEMENTARY DATA

Supplementary Data are available at NAR Online: Supplementary Tables 1–3 and Supplementary Figures 1–3.

## FUNDING

National Science Foundation [MCB-0131782 to I.A.L.-O.]; National Institutes of Health [T32 GM067587 to M.J.L. and P.P.A.]; National Cancer Institute [P30CA014089]. The content is solely the responsibility of the authors and does not necessarily represent the official views of the National Science Foundation, the National Cancer Institute or the National Institutes of Health. Funding for open access charge: I.A.L.-O. donor funds.

*Conflict of interest statement.* None declared.

## Supplementary Material

Supplementary Data
