# Supplementary material for: The role of the C-terminal helix of U1A protein in the interaction with U1hpII RNA
Source: Nucleic Acids Res. 2013 May 22;41(14):7092–100. doi: 10.1093/nar/gkt326 (PMC3737524; doi:10.1093/nar/gkt326)
Supplement: Supplementary Data [file supp_41_14_7092__index.html]

The role of the C-terminal helix of U1A protein in the interaction with U1hpII RNA — The role of the C-terminal helix of U1A protein in the interaction with U1hpII RNA — Supplementary Data 

# The role of the C-terminal helix of U1A protein in the interaction with U1hpII RNA

## Supplementary Data

files

**Files in this Data Supplement:**

- Supplementary Data - pdf file
