# Supplementary material for: The role of the C-terminal helix of U1A protein in the interaction with U1hpII RNA
Source: Nucleic Acids Res. 2013 May 22;41(14):7092–100. doi: 10.1093/nar/gkt326 (PMC3737524; doi:10.1093/nar/gkt326)
Supplement: Supplementary Data [file supp_gkt326_nar-02881-r-2012-File005.pdf]

Supplementary Figures and Tables

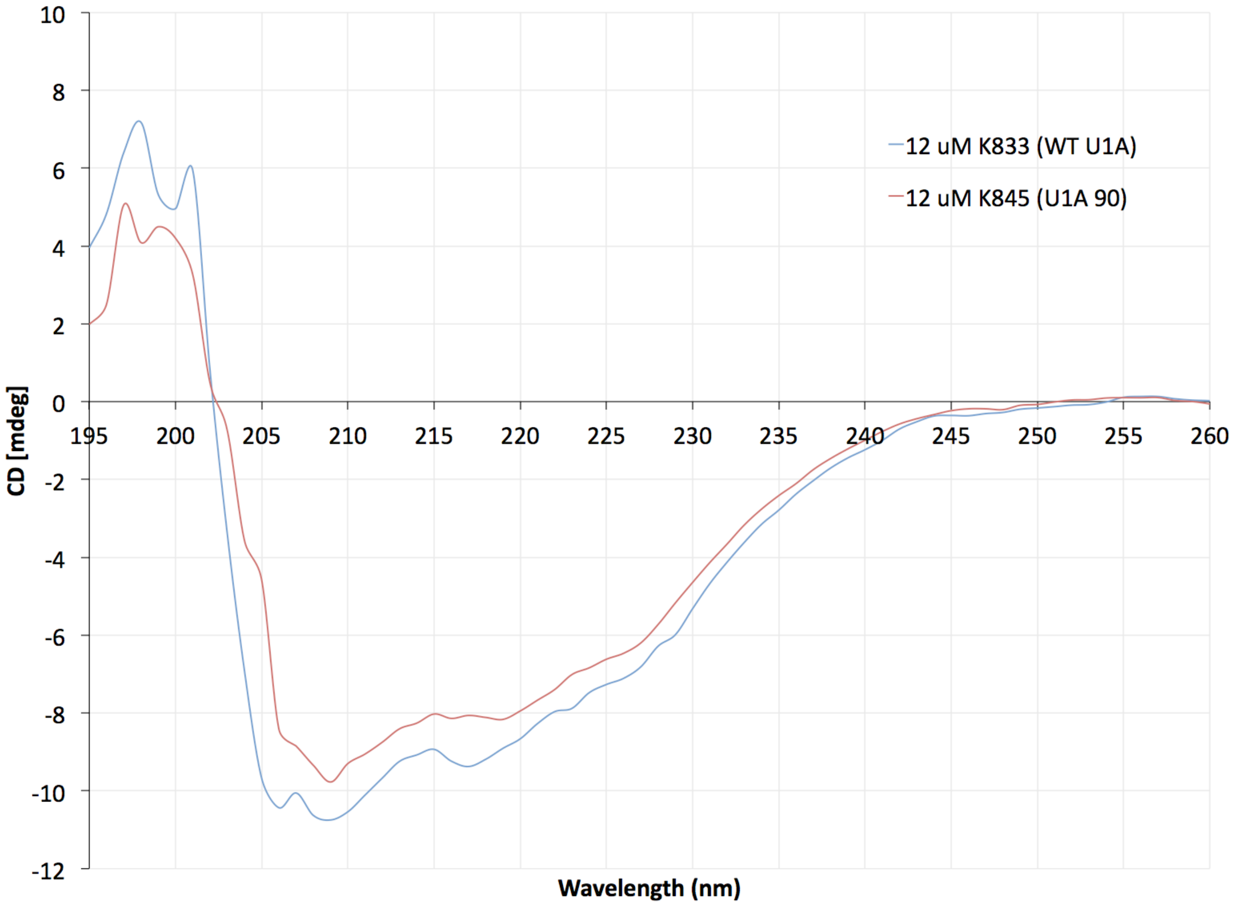

Figure S1. Overlaid CD spectra of U1A (blue) and U1A90 (red). Spectra were recorded at 195 nm - 260 nm from proteins diluted to 12 uM in 10 mM sodium phosphate buffer pH 7.0. The baseline (buffer only) measurement was subtracted from each of the protein spectra before they were overlaid.

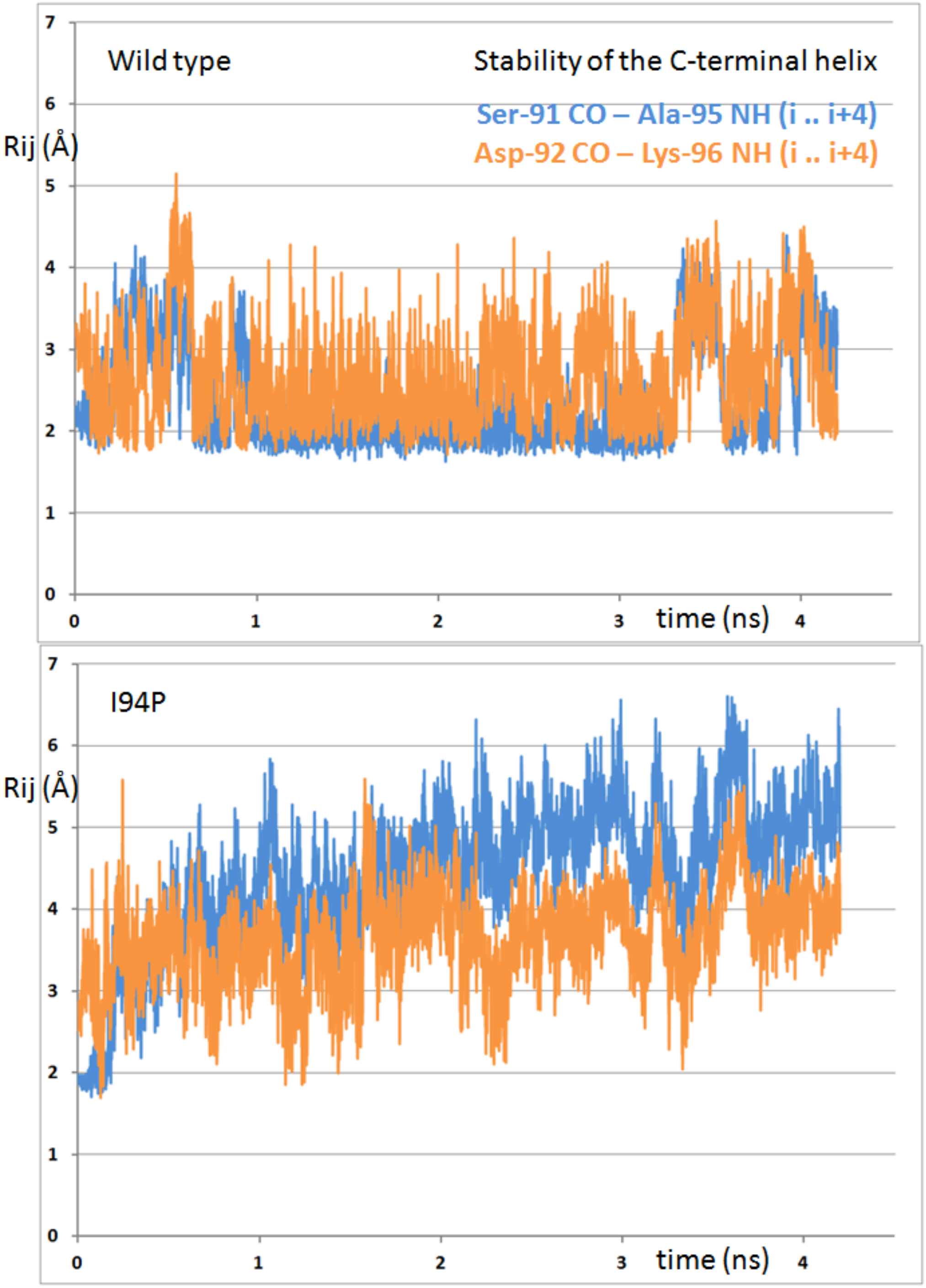

Figure S2. Comparison of intra-helix hydrogen bonds in helix C in brief molecular dynamics simulations of wild-type and Ile94Pro U1A in complex with U1hpII. After only 4 ns, the Ser91-Ala95 and Asp92-Lys96 hydrogen bonds, both of which are required for helix stability, have been disrupted by the Ile94Pro mutation.

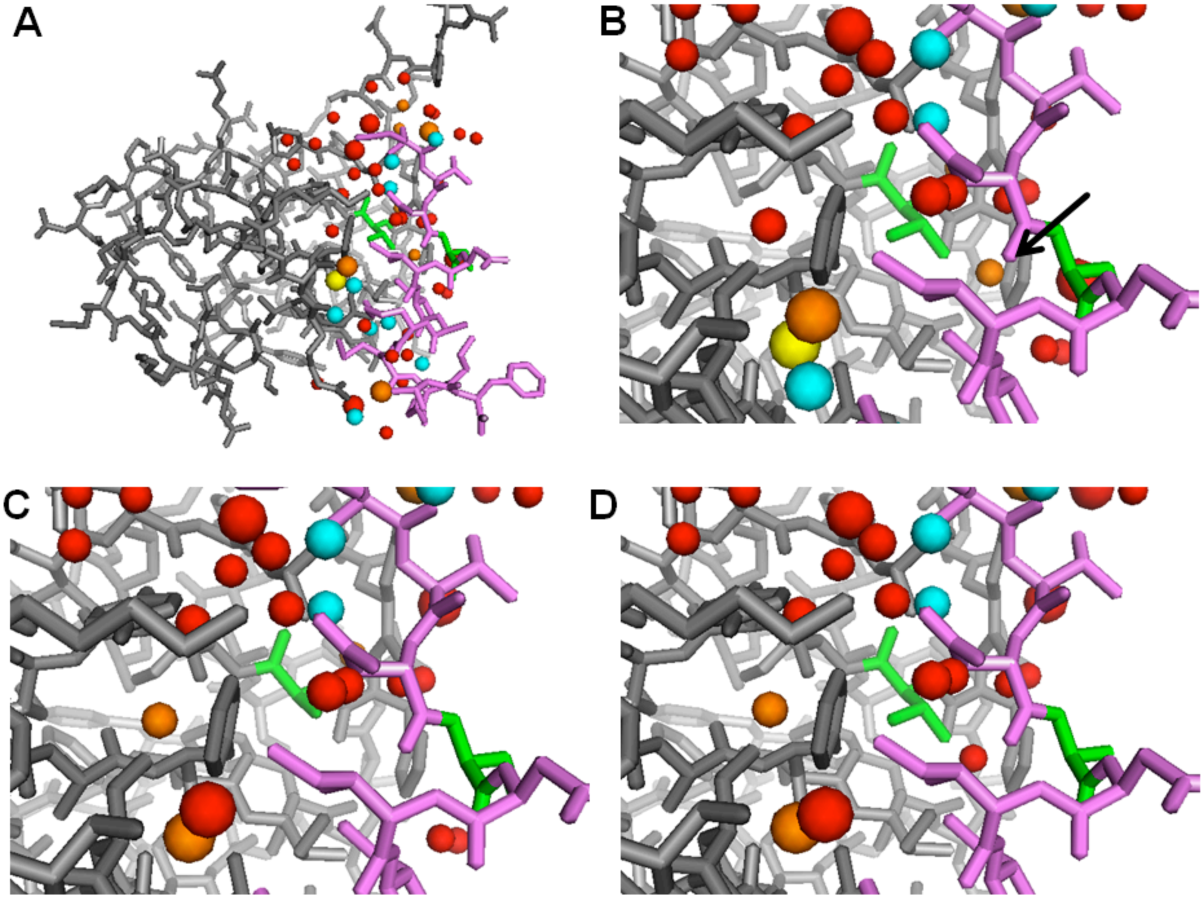

Figure S3. (A) Solvation of helix C (pink) and the RRM (grey) in the free U1A protein. Residues Thr11 (in the RRM) and Ser91 (in helix C) are colored in green. (B) Zoomed in view of the solvation shell in (A), focused on the region around residues Thr11 and Ser91 in wild type U1A. (C) Solvation of this region in the Thr11Ala mutant. (D) Solvation of the same region in the Ser91Ala mutant. Calculations were performed using WATGEN. Water molecules are shown as colored spheres indicating the position of the oxygen atom. Water molecules may be directly hydrogen bonded to bulk water (red), separated from bulk by a single water molecule (orange), or by two water molecules (yellow). Large spheres indicate direct water bridges between helix C and the RRM and small spheres indicate a double-water bridge. Water molecules shown in light blue make a contact with a hydrophobic side chain. The complex shown is number 43 in the ensemble of NMR structures in PDB ID 1FHT (20). This was chosen as a representative complex.

Table S1. Water mediated interactions between Thr11 and Ser91 in the interface between helix C and the RRM in U1A calculated using WATGEN [a]

Model [b]	OG1-OG [c]	OG1-N	OG1-O	N-OG	N-N	N-O	O-OG	O-N	O-O	All
1	0	1	0	0	0	0	0	0	0	1
2	1	0	0	0	0	0	0	0	0	1
3	1	1	0	1	1	0	0	0	0	1
4	1	1	0	0	0	0	0	0	0	1
5	1	1	0	0	0	0	0	0	0	1
6	1	0	0	0	0	0	0	0	0	1
7	0	0	0	0	0	0	0	0	0	0
8	1	1	0	0	0	0	0	0	0	1
9	1	1	0	0	0	0	0	0	0	1
10	1	1	0	1	1	0	0	0	0	1
11	0	1	0	0	1	0	0	0	0	1
12	1	1	1	0	0	0	0	0	0	1
13	1	1	0	0	0	0	0	0	0	1
14	1	1	0	0	0	0	0	0	0	1
15	0	1	1	0	1	0	0	0	0	1
16	1	1	0	0	0	0	0	0	0	1
17	1	1	0	0	1	0	0	0	0	1
18	1	1	0	0	0	0	0	0	0	1
19	0	0	0	0	0	0	0	0	0	0
20	1	0	0	0	0	0	0	0	0	1
21	1	1	0	0	0	0	1	1	0	1
22	1	1	1	0	0	0	0	0	0	1
23	0	1	0	0	0	0	0	0	0	1
24	0	1	0	0	0	0	0	0	0	1
25	0	0	0	0	0	0	0	0	0	0
26	0	0	0	0	0	0	0	0	0	0
27	0	0	0	0	0	0	0	0	0	0
28	1	1	0	0	0	0	0	0	0	1
29	1	1	0	1	1	0	0	0	0	1
30	1	0	0	0	0	0	0	0	0	1
31	0	0	0	0	0	0	0	0	0	0
32	1	1	0	0	0	0	0	0	0	1
33	0	1	0	0	0	0	0	0	0	1
34	1	1	0	0	0	0	0	0	0	1
35	1	1	0	0	0	0	0	0	0	1
36	1	1	0	0	0	0	0	0	0	1
37	1	1	1	0	0	0	0	0	0	1
38	1	1	0	0	0	0	0	0	0	1
39	1	1	0	0	0	0	0	0	0	1
40	1	0	0	0	0	0	0	0	0	1
41	0	0	0	0	0	0	0	0	0	0
42	0	1	0	0	0	0	0	0	0	1
43	1	1	0	0	1	0	0	1	0	1
Total	29	31	4	3	7	0	1	2	0	36

[a] "1" indicates the presence of a water-mediated interaction (single or double water bridge) between the two atoms. In the "All" column, "1" indicates the presence of at least 1 water-mediated interaction between the two atoms.

[b] 43 models in PDB ID 1FHT.

[c] OG1 and OG are hydroxyl oxygen atoms in Thr11 and Ser91 side chains, respectively. N and O indicate backbone atoms. All interactions are shown as Thr11-Ser91.

Table S2. Water-mediated interactions between Ala11 [d] and Ser91 in the interface between helix C and the RRM in U1A (Thr11Ala) calculated using WATGEN [a]

Model [b]	N-OG	N-N	N-O	O-OG	O-N	O-O	All
1	0	0	0	0	0	0	0
2	0	0	0	0	0	0	0
3	0	0	0	0	0	0	0
4	0	0	0	0	0	0	0
5	0	0	0	0	0	0	0
6	0	0	0	0	0	0	0
7	0	0	0	0	0	0	0
8	0	0	0	0	0	0	0
9	0	0	0	0	0	0	0
10	0	0	0	0	0	0	0
11	0	0	0	0	0	0	0
12	0	0	0	0	0	0	0
13	0	0	0	0	0	0	0
14	0	0	0	0	0	0	0
15	0	1	0	0	0	0	1
16	0	0	0	0	0	0	0
17	0	0	0	0	0	0	0
18	0	0	0	0	0	0	0
19	0	0	0	0	0	0	0
20	0	0	0	0	0	0	0
21	0	0	0	0	0	0	0
22	0	0	0	0	0	0	0
23	0	0	0	0	0	0	0
24	0	0	0	0	0	0	0
25	0	1	0	0	1	0	1
26	0	0	0	0	0	0	0
27	0	0	0	0	0	0	0
28	0	0	0	0	0	0	0
29	0	0	0	0	0	0	0
30	1	0	0	0	0	0	1
31	0	0	0	0	0	0	0
32	0	0	0	0	0	0	0
33	0	0	0	0	0	0	0
34	0	0	0	0	0	0	0
35	1	1	0	0	0	0	1
36	0	0	0	0	0	0	0
37	0	0	0	0	0	0	0
38	0	0	0	0	0	0	0
39	0	0	0	0	0	0	0
40	0	0	0	0	0	0	0
41	0	0	0	0	0	0	0
42	0	0	0	0	0	0	0
43	0	1	0	0	1	0	1
Total	2	4	0	0	2	0	5

[a,b] As for Table S1

[c] OG is the hydroxyl oxygen atom in the Ser91 side chain. N and O indicate backbone atoms. All interactions are shown as Ala11-Ser91.

[d] The Thr11 to Ala11 mutations was made using AMBER10, with a very brief constrained minimization that did not alter the structure of the protein.

Table S3. Water-mediated interactions between Thr11 and Ala91 [d] in the interface between helix C and the RRM in U1A (Ser91Ala) calculated using WATGEN [a, b]

Model [b]	OG1-N	OG1-O	N-N	N-O	O-N	O-O	All
1	1	0	0	0	0	0	1
2	0	0	0	0	0	0	0
3	1	0	0	0	0	0	1
4	0	0	0	0	0	0	0
5	1	1	0	0	0	0	1
6	0	0	0	0	0	0	0
7	0	0	0	0	0	0	0
8	1	0	0	0	0	0	1
9	0	0	0	0	0	0	0
10	1	0	0	0	0	0	1
11	1	0	1	0	0	0	1
12	1	0	1	0	0	0	1
13	0	0	0	0	0	0	0
14	1	1	0	0	0	0	1
15	1	0	0	0	0	0	1
16	1	0	0	0	0	0	1
17	1	0	1	0	0	0	1
18	0	0	0	0	0	0	0
19	0	0	0	0	0	0	0
20	0	0	0	0	0	0	0
21	1	0	0	0	0	0	1
22	1	0	0	0	0	0	1
23	1	0	0	0	0	0	1
24	1	0	0	0	0	0	1
25	0	0	0	0	0	0	0
26	0	0	0	0	0	0	0
27	0	0	0	0	0	0	0
28	0	0	0	0	0	0	0
29	0	0	0	0	0	0	0
30	0	0	0	0	0	0	0
31	0	0	0	0	0	0	0
32	1	0	0	0	0	0	1
33	1	0	0	0	0	0	1
34	0	0	0	0	0	0	0
35	1	0	0	0	0	0	1
36	1	0	0	0	0	0	1
37	1	0	0	0	0	0	1
38	1	0	0	0	0	0	1
39	0	0	0	0	0	0	0
40	1	0	1	0	0	0	1
41	0	0	0	0	0	0	0
42	1	0	1	0	1	0	1
43	1	0	0	0	0	0	1
Total	24	2	5	0	1	0	24

[a,b] As for Table S1

[c] OG1 is the hydroxyl oxygen atom in the Thr11 side chain. N and O indicate backbone atoms. All interactions are shown as Thr11-Ala91.

[d] The Ser91 to Ala91 mutations was made using AMBER10, with a very brief constrained minimization that did not alter the structure of the protein.
